# Traumatic abdominal wall hernias: disruptions of the abdominal wall muscles associated to pelvic bone fractures illustrated by two case reports

**DOI:** 10.1186/s12893-020-00909-2

**Published:** 2020-10-27

**Authors:** Leïlani Delaune, Sylvain Steinmetz, Hafize Heutschi-Öztürk, Olivier Borens

**Affiliations:** Centre Hospitalier Universitaire Vaudois, University of Lausanne, Lausanne, Switzerland

**Keywords:** Traumatic abdominal wall hernia, Abdominal wall disruption, Pelvic fractures, Blunt abdominal trauma

## Abstract

**Background:**

Blunt abdominal traumas are often associated with intra-abdominal injuries and pelvic fractures. Traumatic abdominal wall hernias due to disruption of the abdominal wall muscles may be overlooked. Delayed diagnosis can lead to hernia related complications.

**Case presentation:**

We present two cases of high kinetic trauma with pelvic fractures and acute traumatic abdominal wall herniation. Both of these cases suffered from a delayed diagnosis and needed surgery to treat the symptomatic herniation.

**Conclusion:**

Clinical reassessment and appropriate medical imaging are mandatory in patients with high kinetic abdominal blunt traumas and associated pelvic fracture, in order to prevent delayed diagnosis and possible complications.

## Background

Blunt abdominal trauma often occurs in road accidents, occasionally leading to major pelvic and intra-abdominal injuries [1, 2]. Patients admitted to the emergency room undergo a physical examination according to the ATLS® guidelines, in order to assess and manage vital injuries.

However, among the multiple lesions found in a poly-trauma patient, abdominal wall hernias due to the disruption of the abdominal wall muscles are frequently missed. These traumatic abdominal wall hernias may not contain digestive structures, but can be a muscle laceration causing a weakness in the abdominal wall. This may later lead to an intestinal incarceration. Delayed diagnosis leads to prolonged suffering and the need for complex surgical procedures.

Traumatic lumbar or abdominal wall hernias combined with a pelvic fracture have scarcely been described in the literature. Fractures of the pelvis are serious and life-threatening injuries, usually caused by a high energy trauma, frequently leading to disruption of the abdominal wall muscles [2–4].

Surgical reconstruction is necessary to strengthen the abdominal wall and reduce the risk of hernia related complications.

Given the paucity of reported cases we hereby present a review of the literature on pelvic fractures and associated disruptions of the abdominal wall muscles, after describing two cases of delayed diagnosis and treatment of abdominal wall muscle disruption following a high energy trauma.

## Case presentation

### Case 1

In 2016, a 46-year-old patient, involved in a car-on-car accident was admitted to the emergency department. The patient was driving the vehicle and had his belt on. At admission, the hemodynamics were stable, the Glasgow Coma Scale (GCS) was 15 and the Injury Severity Score (ISS) was of 12 points. Physical and radiographic examination (standard X-ray and a trauma CT with contrast) showed a lateral compression fracture of the pelvis with a displaced comminuted fracture of the left iliac crest, bilateral pulmonary contusions, and a disruption of several abdominal wall muscles with an iliac muscle hematoma.

No surgery was performed, and the patient was authorized to walk full weight-bearing as tolerated. The patient had a follow up every 6 months, had little and bearable abdominal pain, and never had any intra-abdominal problem.

**Fig. 1:**
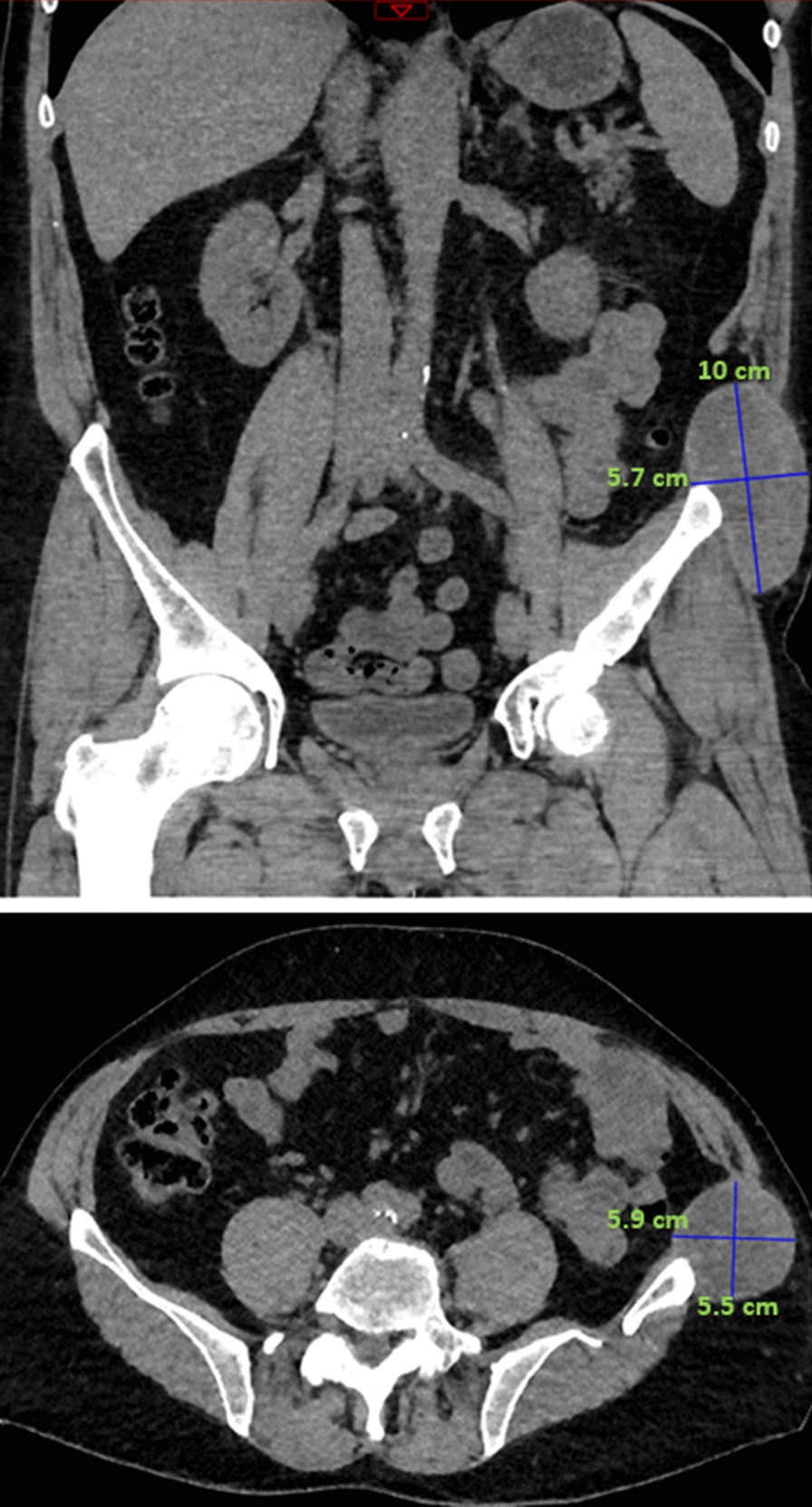
CT abdominal scan: parietal collection near the left iliac crest

At 2-year follow up, the patient had persistent and constant abdominal pain in the left groin, radiating down the left thigh, which had increased in the last few months. An abdominal and pelvic CT with contrast was performed, showing a 10 cm parietal collection near the left iliac crest with disruption of the left internal oblique muscle and the left transverse muscle (Fig. 1).

**Fig. 2:**
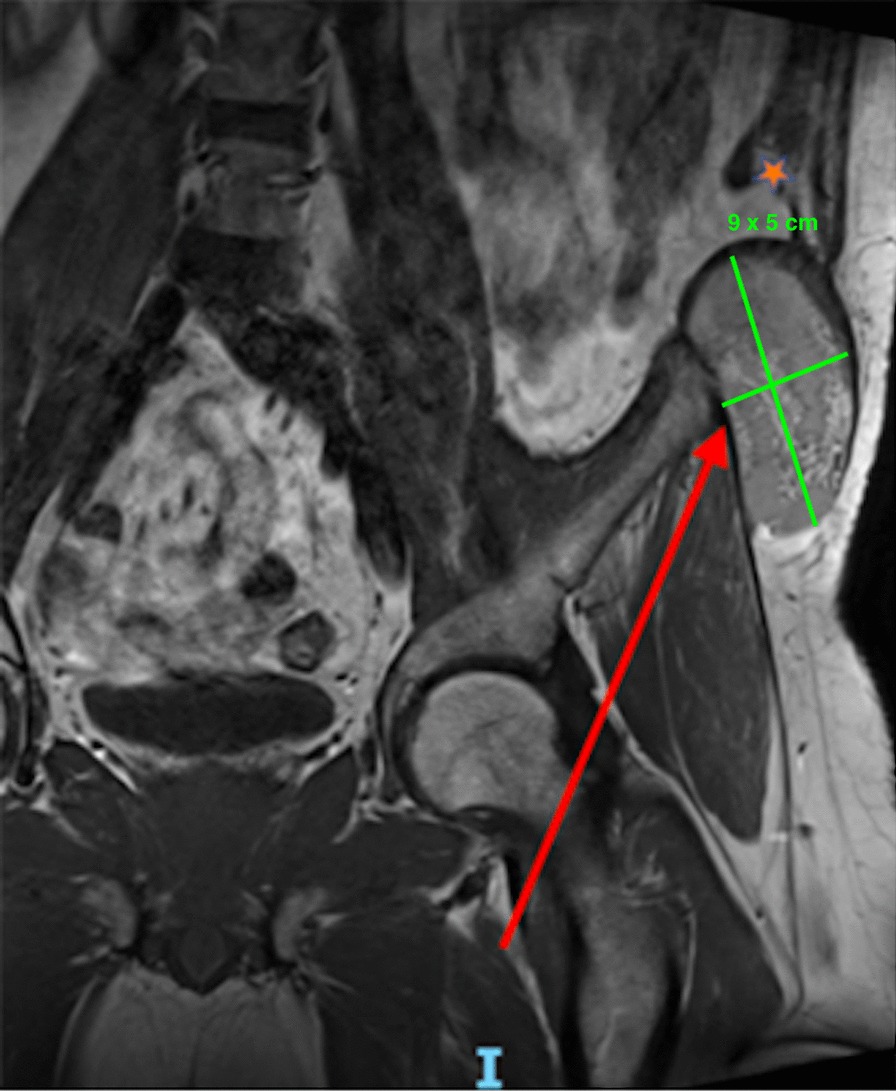
Coronal MRI views in T2 signal: red arrow: heterogeneous parietal collection, 9 × 5 cm; Orange star: internal oblique’s free edge, 7 cm from the iliac crest

MRI was performed, characterizing the collection as partially hemorrhagic, and located between the oblique and the transverse muscle, the tensor fasciae latae and the gluteus medius. The distance between the left iliac crest and the internal obliques’ free edge was 7 cm (Fig. 2).

The patient requested surgery to treat the chronic persistent abdominal pain. The surgeon approved the surgical indication, the patient being at risk for an incarceration of intra-abdominal organs due to the weakness in the abdominal wall.

### Case 2

**Fig. 3:**
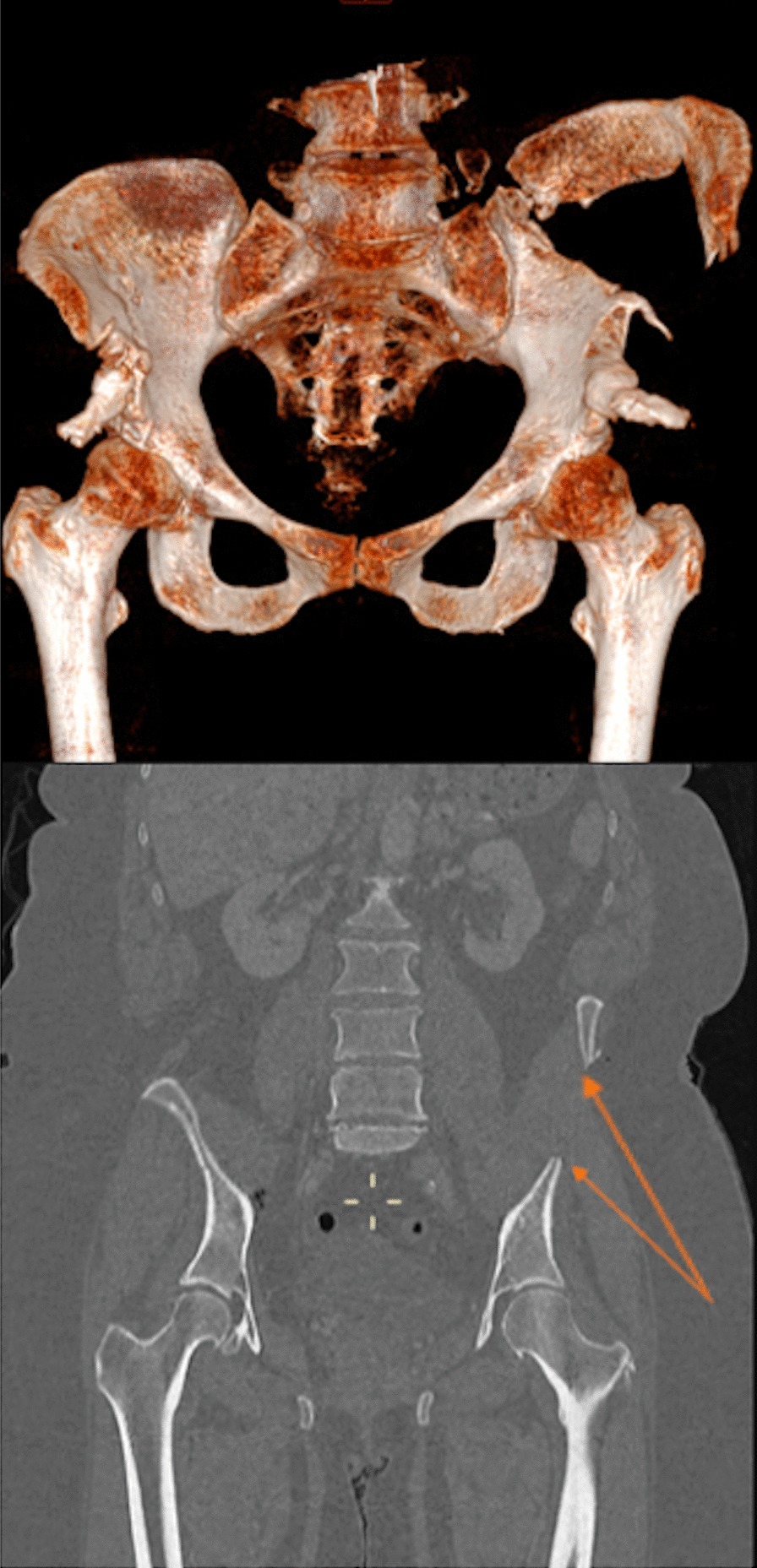
Abdominal CT scan: fracture of the left iliac crest (arrow)

A 57-year-old patient, car passenger, involved in a high-speed car-on-car accident in 2017, was admitted to the emergency department. The patient had stable hemodynamics, had a GCS of 14 and the ISS was of 12 points. Initial assessment showed multiple osseous lesions, including a lateral compression fracture of the pelvic ring with a comminuted bilateral fracture of the iliac crests, left costal and clavicle fractures, a manubrium sterni fracture associated with an anterior mediastinal hematoma, fractures of the transverse processes from L1 to L5, a L2–L3 plexopathy with a motor and sensitive deficit of the femoral nerve and an open Gustilo II left Weber C ankle fracture (Fig. 3).

The costal fractures, clavicle fracture and left ankle fracture were surgically treated. The pelvic fractures were treated conservatively, and full weight bearing as tolerated was authorised, with a cast on the left leg, for 6 weeks.

**Fig. 4:**
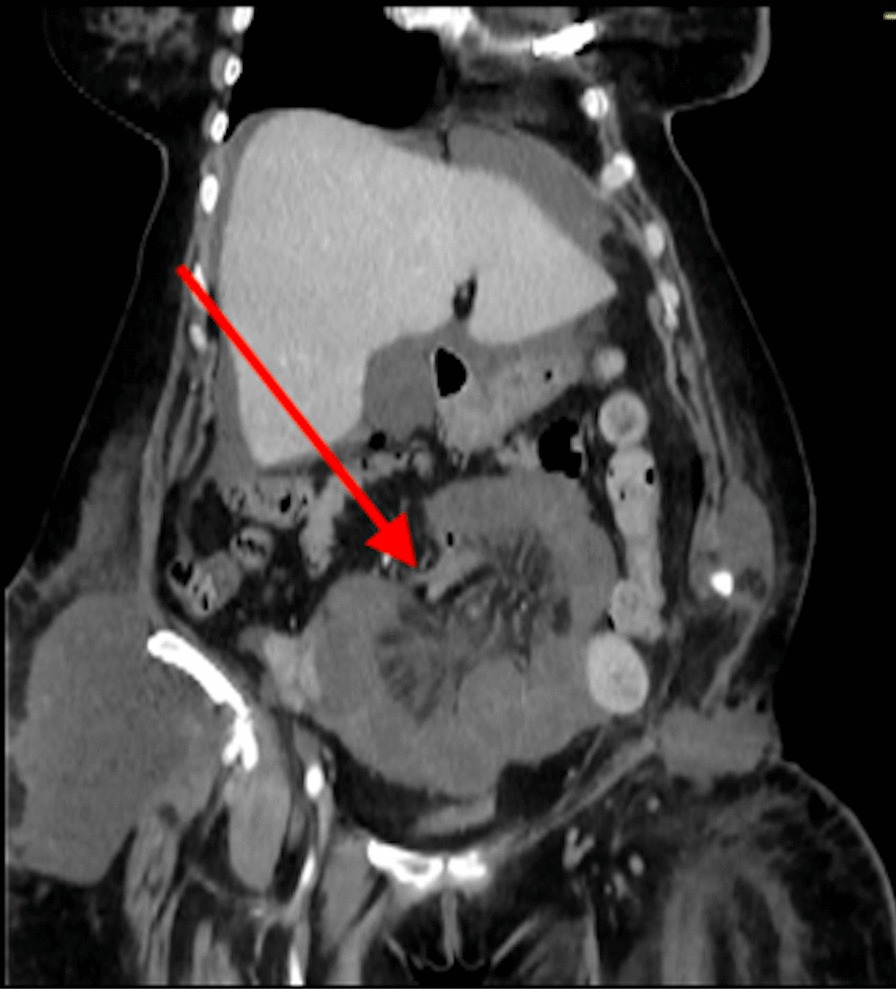
Abdominal CT scan: coronal views. Internal hernia and beginning of bowel ischemia (arrow)

A month later, the patient suffered from brutal and intense abdominal pain. An internal hernia was diagnosed on an abdominal CT scan (Fig. 4). The collar of the hernia measured 7–8 cm, and the sac contained the sigmoid colon. The hernia was surgically reduced, and a 26 × 36 cm prosthetic monofilament polypropylene mesh was put in place, fixed from the iliac crest to the subinguinal hiatus and to the anterior space of the left kidney.

A year later, the patient still suffered from little abdominal pain, mainly during bowel movement. Bilateral abdominal lumps were palpable during clinical examination. The patient also complained about abnormal movement or "clicking" at the level of the left iliac crest fracture.

**Fig. 5:**
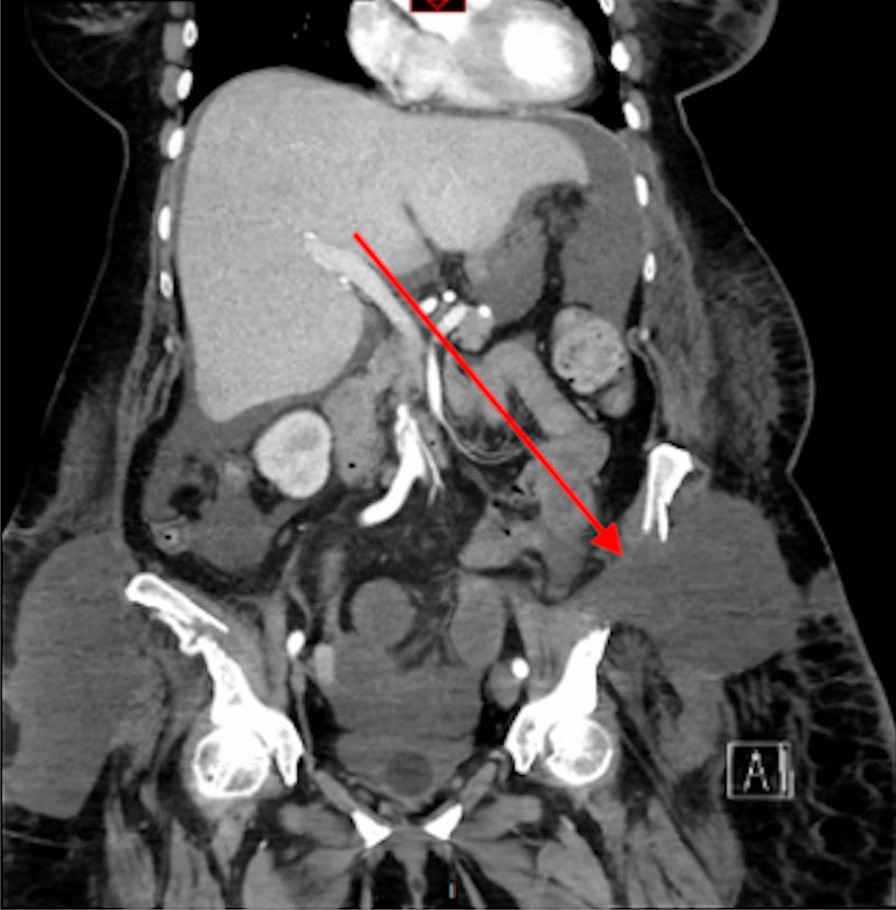
Abdominal CT scan: interfragmentary hernia (arrow)

An abdominal CT scan was done, showing an important defect in the anterior abdominal wall. A non-union of the left iliac crest was also shown, with an interfragmentary hernia (Fig. 5). An asymptomatic right paravertebral hernia was also visible. The patient was still treated conservatively, with physiotherapy sessions, in order to reinforce the abdominal wall and paraspinal muscles. The follow up occurred every 6 months.

In 2019, the patient had a perfectly normal clinical exam, and was free from pain.

## Discussion and conclusion

Traumatic abdominal wall hernias linked to pelvic fractures have rarely been described [2, 4, 5], even though they can be encountered in our everyday practice. They are defined as a bowel or abdominal organ herniation through a disruption of the musculature and fascia following adequate trauma [6], with no skin penetration or pre-existing hernia [7]. The muscles that make up the abdominal wall are all attached to the pelvic bone.

Abdominal wall hernias may occur whenever there is a disruption of the abdominal wall muscles following blunt abdominal trauma, even more so if a pelvic fracture is associated. Pelvic fractures often occur in high energy trauma, such as in road accidents. They are often associated with several intra-abdominal injuries, or other life-threatening injuries.

Traumatic abdominal wall hernias were first described by Selby in 1906, concluding the fact that muscles and fasciae were lacerated, whilst the integument and peritoneum remained unharmed [8].

However, in 1932, Moorhead described true traumatic abdominal wall hernias as being a myth [9, 10], as he stated that an isolated or single injury is never the source of origin of inguinal hernia.

However, several mechanisms of traumatic abdominal wall hernias have since been described for example, the “seat belt hernia” [11, 12] is when the seat belt causes a massive disruption of the subcutaneous fat and rectus muscle in a high energy trauma. The “handlebar hernia" [11, 13] occurs in motorcycle, bicycle or snowmobile accidents, causing a tear of the underlying abdominal muscle and fascia without necessarily disrupting the skin. This massive disruption phenomenon is due to local tangential shearing forces associated to the rise of intra-abdominal pressure during the blunt abdominal trauma.

Most of the traumatic abdominal wall hernias described through case reports stated a delay of presentation, mainly due to other life-threatening injuries, requiring urgent medical attention, as in our two cases. Furthermore, some articles described a possible muscle reaction that may mask an abdominal wall defect, following the trauma [14]. Some reported that the abdominal wall was certainly weakened due to the development of a haematoma or wound infection in the trauma’s aftermath, leading to a delayed herniation [15].

Performing a CT scan is crucial in the diagnosis of a traumatic abdominal wall hernia, not only in the primary evaluation after a blunt abdominal trauma, but also in the aftermath. It can assess for a possible disruption of the abdominal wall which may lead to a hernia. A grading of abdominal wall disruptions on CT scans was described by Dennis et al. in 2009 [16], ranging from a simple subcutaneous tissue contusion to a complete abdominal wall disruption with evisceration (Table 1).

**Table 1:** Grading of abdominal wall disruptions on CT scan (Dennis et al. 2009) [16]

Grade	Description
I	Subcutaneous tissue contusion
II	Abdominal wall muscle hematoma
III	Singular abdominal wall muscle disruption
IV	Complete abdominal wall muscle disruption
V	Complete abdominal wall muscle disruption with herniation of the abdominal contents
VI	Complete abdominal wall disruption with evisceration

The use of an MRI may also be useful to evaluate which abdominal wall muscles are disrupted, and the distance between the two stumps.

According to the Young and Burgess classification, traumatic abdominal wall hernias are most often associated to lateral compression fractures of the pelvis. Other pelvic fractures may nevertheless also be associated with abdominal wall hernias: one report for example described an open book pelvic fracture presenting with an inguinal hernia [14].

Conservative treatment is often chosen in lateral compression pelvic fractures. However, a possible tearing mechanism may occur during trauma, with contralateral disruption of the abdominal wall muscles, as is described in our cases. Through analysing cadaveric human abdominal muscles, the external and internal oblique muscles have a compromised ability to generate active force when the spine is laterally bent to the contralateral side [17].

Several reports recommended an early abdominal exploration and repair of the hernia with surgical mesh, due to a possible and potential incarceration [18, 19]. Furthermore, an internal fixation of the pelvic fracture (though often stable in lateral compression fractures) may be a relative indication in blunt abdominal traumas, as these are often associated with acute traumatic abdominal wall hernias. Surgery is however not innocuous, and can lead to several complications, such as infections, chronic pain, or digestive fistulae.

As described in our report, the delay in presentation of traumatic abdominal wall hernias often leads to a delayed repair. In our cases, the decision to repair was based on the patients’ symptoms. However, the surgical treatment could and should have been discussed earlier. Both our patients presented a post-traumatic weakness in the abdominal wall, making them at risk for having a hernia, with or without digestive tract content, thus at risk for an incarceration of intra-abdominal organs.

The debate on repairing traumatic abdominal wall hernias associated with pelvic fractures mainly depends at the time of diagnosis, on other associated injuries, and the patients’ clinical symptoms.

It is important to clinically reassess the patient after a blunt abdominal trauma throughout the various stages of the patient’s management to detect a possible traumatic abdominal wall hernia and optimise the medical and surgical care.

Traumatic abdominal wall hernias are rarely diagnosed in patients with pelvic fractures. Patients with pelvic fractures after high energy trauma are often subject to several other injuries diagnosed during the immediate and delayed management, and disruptions or avulsions of the muscles of the abdominal wall can be missed. Further studies must be conducted in order to obtain a systematic pattern of physical examination for patients presenting with pelvic fractures, in the acute and chronic stage of the trauma, to include the diagnosis of traumatic abdominal wall disruption.

## Data Availability

The authors declare that the data supporting the findings of this study are available within the article.

## Abbreviations

GCS: Glasgow Coma Scale
ISS: Injury Severity Score
